
JOURNAL INFORMATION
==============================
NLM Title Abbreviation: Dent Regist
NLM Journal ID: 0006672

The Dental Register

Publisher: Biomedical Journal Digitization Project

ARTICLE INFORMATION
==============================
PMCID: PMC6916912
PMID: 33698198
Article version: 1
Subjects: Editorial

Important Decision

Print publication date: 1884 Jun
Volume: 38
Issue: 6
First page: 316
Last page: 316
License: This work is free of known copyright restrictions. For more information, please see PMC Back Issue Digitization.
License URL: https://creativecommons.org/publicdomain/mark/1.0/

==============================

### Important Decision.

The Illinois State Board of Dental Examiners, at their regular meeting over a year ago, refused to recognize a diploma from the Indiana Dental College as sufficient to entitle the holder to practice dentistry in that State. The board refused to register such person without examination. Suit was brought against the board to compel such recognition. The case passed in regular order to the Supreme Court of the State.

Within a few days a decision has been issued from that court fully sustaining the Board of Examiners in the position they took in the matter.

It is a matter of regret that any dental college will refuse to assume a position that will command due recognition; at least throughout our own country, especially at the hands of examining boards who act under sanction of law.

### Meeting.

The Twenty-Sixth Annual Meeting of the Indiana State Dental Association will be held at Indianapolis, commencing Tuesday, June 24, 1884. The meeting promises to be one of great interest. Quite a number of dentists from other States are expected to be present; among others will be Dr. W. Sudduth, of Ill., who will read a paper and give illustrations on "Dento Embryonal Histology." A cordial invitation is extended to all.
